# Does a pre-hospital emergency pathway improve early diagnosis and referral in suspected stroke patients? – Study protocol of a cluster randomised trial [ISRCTN41456865]

**DOI:** 10.1186/1472-6963-5-66

**Published:** 2005-10-11

**Authors:** Marica Ferri, Assunta De Luca, Paolo Giorgi Rossi, Giuliano Lori, Gabriella Guasticchi

**Affiliations:** 1 Agenzia di Sanità Pubblica della Regione Lazio, Via di Santa Costanza, 53, 00198 Roma, Italy

## Abstract

**Background:**

Early interventions proved to be able to improve prognosis in acute stroke patients. Prompt identification of symptoms, organised timely and efficient transportation towards appropriate facilities, become essential part of effective treatment. The implementation of an evidence based pre-hospital stroke care pathway may be a method for achieving the organizational standards required to grant appropriate care. We performed a systematic search for studies evaluating the effect of pre-hospital and emergency interventions for suspected stroke patients and we found that there seems to be only a few studies on the emergency field and none about implementation of clinical pathways.

We will test the hypothesis that the adoption of emergency clinical pathway improves early diagnosis and referral in suspected stroke patients. We designed a cluster randomised controlled trial (C-RCT), the most powerful study design to assess the impact of complex interventions. The study was registered in the Current Controlled Trials Register: ISRCTN41456865 – Implementation of pre-hospital emergency pathway for stroke – a cluster randomised trial.

**Methods/design:**

Two-arm cluster-randomised trial (C-RCT). 16 emergency services and 14 emergency rooms were randomised either to arm 1 (comprising a training module and administration of the guideline), or to arm 2 (no intervention, current practice). Arm 1 participants (152 physicians, 280 nurses, 50 drivers) attended an interactive two sessions course with continuous medical education CME credits on the contents of the clinical pathway. We estimated that around 750 patients will be met by the services in the 6 months of observation. This duration allows recruiting a sample of patients sufficient to observe a 30% improvement in the proportion of appropriate diagnoses.

Data collection will be performed using current information systems. Process outcomes will be measured at the cluster level six months after the intervention. We will assess the guideline recommendations for emergency and pre-hospital stroke management relative to: 1) promptness of interventions for hyperacute ischaemic stroke; 2) promptness of interventions for hyperacute haemorrhagic stroke 3) appropriate diagnosis. Outcomes will be expressed as proportions of patients with a positive CT for ischaemic stroke and symptoms onset <= 6 hour admitted to the stroke unit.

**Discussion:**

The fields in which this trial will play are usually neglected by Randomised Controlled Trial (RCT). We have chosen the Cluster-randomised Controlled Trial (C-RCT) to address the issues of contamination, adherence to real practice, and community dimension of the intervention, with a complex definition of clusters and an extensive use of routine data to collect the outcomes.

## Background

Stroke is the third most common cause of death in developed countries [1]. In 80% of cases stroke is ischaemic (caused by thrombotic or embolic occlusion of cerebral artery) [2] the remainder are caused by intracerebral or subarachnoid haemorrages.

Around 10% of all people with acute ischaemic stroke will die within 30 days of stroke onset, while 50% of the survivors will experience some level of disability after 6 months [3].

As early effective interventions proved to be able to improve prognosis [1], implementation of an evidence based pre-hospital stroke care pathway may be a method for achieving early identification of symptoms, and organised timely and efficient transportation towards appropriate care. Clinical pathways have been indicated with different names: care pathways, critical pathway, critical path method, and Care Maps(tm) [4]. We adopted "clinical pathway" to indicate a document targeted to all emergency service personnel (dispatchers, physicians, nurses and any other professionals, such as ambulances drivers) involved in the management of a suspect acute stroke from pre-hospital to emergency phases.

A systematic review on the adoption of in-hospital clinical pathways suggested that the currently available evidence is insufficient to support routine implementation of care pathways for the hospital management of acute stroke or stroke rehabilitation [5]. Therefore there is still uncertainty about the effectiveness of clinical pathways to achieve earlier interventions for stroke patients in pre-hospital setting. We are performing a systematic review on the effectiveness of clinical pathways in pre-hospital field and we also decided to design the present study. The results of the study will be the basis to decide about the widespread implementation of the clinical pathway in the whole region.

**Figure 1:**
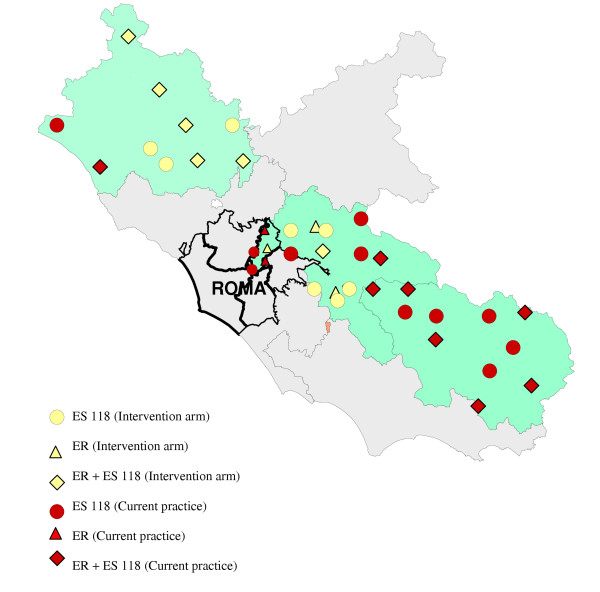
Territorial distribution of the entities enclosed in the study.

Within Lazio, the region with 5,302,302 inhabitants in which the capital city of Rome is located, the emergency medical services (ES) are accessible by dialling "118" with automatic connection to the nearest dispatch facility. The emergency network is organised in three levels of complexity. The basic level involves emergency rooms for first aid; the first level is equipped for mild-severe diagnosis and the second level for the most severe urgency. These services are in a number and a geographical disposition to grant the required assistance in emergency (see figure 1). Calls for interventions are answered by ES dispatchers who activate the nearest ambulance unit. The Basic Life Service (BLS) facilities are superior in number than the Advanced Life Services (ALS) and are commonly forwarded for the suspected stroke patients. Nowadays, BLS are compelled by law to deliver patients to the nearest emergency hospital. There, medical doctors provide the assessment of patients conditions and diagnosis and decide about referral to appropriate facility. The provision of Computerised Tomography (CT) to confirm the diagnosis of stroke, and the transportation of patients to their final destination, may require some times. With the actual organization of system, the process from the emergency call to the admission to the stroke unit may take from several hours up to days.

The availability of effective early interventions for stroke, calls for reengineering these procedures.

The need for prompt diagnosis and referral requires a that more responsibility is placed on nurses (active in the BLS) who should be trained to recognize early symptoms and should be empowered to contact ES in order to identify the appropriate level of care required, and to transport patients directly to stroke units.

An evidence-based clinical pathway was developed with the methods adopted by the most qualified guidelines development agencies (such as Scottish Intercollegiate Guidelines Network, National Institute for Clinical Excellence and the Italian National Plan for Guidelines Development-PNLG) with the involvement of the emergency health workers in the expert panel, and we decided to evaluate its effectiveness in standardising and improving the procedures in pre-hospital (ES) and emergency (ER) setting, before considering its widespread adoption in our region.

The cluster randomised trial design is indicated in assessing the effectiveness of complex interventions [6] and it allows comparisons of alternative strategies such as guidelines versus conventional educational methods of influencing doctors' management of a particular problem. In our study randomization at cluster level is not only indicated but also necessary to avoid hindrance to the normal activity of the services. We, therefore, adopted as unit of randomization the whole teams of ER or ES stations to avoid contamination.

We present the protocol of study that compares the adoption of an evidence-based prehospital pathway versus current practice.

It is our intention to describe the methodology of the study in the present publication in order to ensure independence of the results and to stimulate criticism and suggestions from the journal readers. The protocol has been presented in several international conferences to share our initiative with the scientific community and to collect their comments and ideas.

As we are aware of the many limits and peculiarity of this study design we believe that lessons on how to deal with complex intervention in the difficult environment will be the added value of the present study.

## Methods/design

### Participants

**Figure 2:**
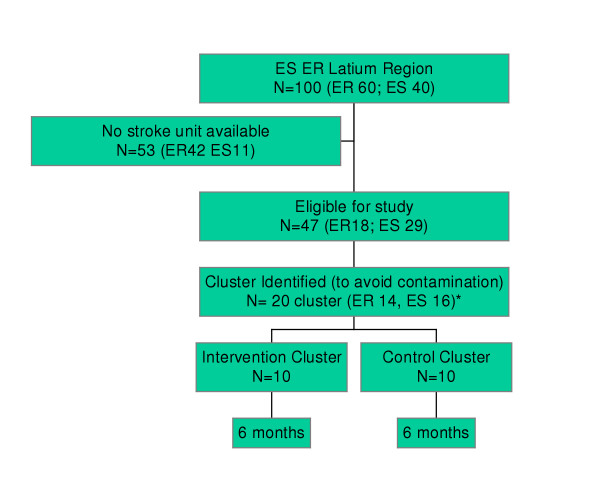
**Flow chart of randomization procedure. **Overall we had 20 units of randomization (cluster) of which 10 are couples composed of one ES+1ER on the basis of closeness (usually the ES is situated in the same place of the ER, or systematically refers patients in the same closest ER); 6 are ES and 4 are ER which could not be matched.

We identified all the emergency services referring to the two stroke units presently available in Rome (figure 1). There were 47 entities comprising 18 emergency rooms and 29 emergency service stations (52 ambulances) from which we created 20 clusters. The criteria for cluster creation was grouping together the services sharing personnel and/or referring patients each other (figure 2-table 1).

**Table 1:** Availability of ambulances and organization of structures in the study area, as reported April 2005.

**Place/Area**	**Emergency Medical Services (ES)**	**Emergency room (ER)**	**Notes**
**Viterbo**			
Viterbo	*Call Center *with Medical Doctors	ER level I	
Montalto di Castro	Medical doctors (h12 closed for holidays)	-	
Tarquinia	Only nurses	ER first aid	Staff sharing ES/ER
Tuscania	Only nurses (h12)	ER first aid	
Vetralla	Medical doctors	-	
Ronciglione	Only nurses	-	Staff sharing ES/ER
Civita Castellana	Only nurses	ER first aid	Staff sharing ES/ER
Orte	Medical doctors	-	
Monte Fiascone	Only nurses	ER first aid	Staff sharing ES/ER
Acquapendente	Medical doctors	ER first aid	Staff sharing ES/ER
**ASL FR**			
Anagni	1 full staffed ambulance + 1 BLS	ER first aid	
Alatri	1 full staffed ambulance + 1 BLS	ER first aid	
Fiuggi	1 ALS – ambulances with medical doctors (4)	-	
Ferentino	1 ALS – ambulances with medical doctors (8)	-	
Frosinone	*Call Center *with medical doctors(8)	ER level I	
Ceccano	2 BLS	ER first aid	
Veroli	1 BLS	-	
Ceprano	2 BLS	-	
Sora	1 full staffed ambulance + 1 BLS	ER first aid	
Isola liri	2 BLS	-	
Atina	1 ALS – ambulance with medical doctor (5)	-	
Pontecorvo	1 First level (staffed for most severe conditions)+ 1BLS	ER first aid	
Cassino	2BLS (only inter-hospital transfer)	ER level I	
**ASL RMG**			
Tivoli	2 BLS	ER level I	A new ER will be opened (during the study period)
Monterotondo	1 BLS	ER first aid	ES and ER randomized independently
Palombara	1 BLS	-	
Palestrina	1 BLS	ER first aid	ES and ER randomized independently
Colleferro	1 full staffed ambulance(h12) + 2 BLS	ER first aid	ES and ER randomized independently
Valmontone	1 BLS	-	
Subiaco	1 BLS	ER first aid	
Olevano Romano	1 BLS	-	
Lunghezza	1 ALS – ambulances with medical doctors (4)	-	
Arsoli	1 BLS	-	
Montelanico	1 BLS	-	
**ASL RMA**			
S. Giacomo	full staffed ambulance – ambulances with medical doctors	ER level I	ES and ER randomized independently
Nomentano	Red Cross (as ALS)	-	
Arno-Treviso	3 BLS + 1 full staffed ambulance – ambulances with medical doctors	-	
Addolorata	3 BLS + 1 ALS – ambulances with medical doctors	-	
Marcigliana	1 BLS	-	

Participants are all the workers belonging to the services enclosed in the study (enclosing the ambulances' drivers).

About 152 physicians, 280 nurses, 50 drivers will be trained on the contents of the clinical pathway. We estimated that around 750 patients will be met by the services in the 6 months of observation.

### Interventions

Participants in the intervention arm will be trained on the content of the clinical pathway and will be given the clinical pathway itself for consultation and discussion in groups.

The clinical pathway is based on available evidence based pre-hospital and emergency interventions for suspected stroke patients.

It consists of the following main points:

- ES dispatcher uses a short form of Cincinnati pre-Hospital Stroke Scale (CHSS) to identify suspected stroke patients during the telephone call;

- ES health workers confirm diagnosis by CHSS on the scene;

- patients are provided CT and referred to appropriate care.

A group of trainers from the expert panel that developed the clinical pathway, and composed of:

- an ES medical doctor

- anaesthesiologist working on helicopter emergency unit

- two neurologists working in stroke unit

- a physician working in an emergency department

- a neurosurgeon

- two epidemiologist and evidence-based medicine expert

trained a group of health workers selected from all the entities participating in the intervention arm study to act as "facilitators" for peer education. The facilitators trained their colleagues in the workplace with the help of audiovisual materials produced by the teachers themselves.

At least one representative of the teachers' group took part in the meetings on the workplace to support groups' discussion and to answer possible questions. As a result of the training sessions every person working in any entity participating in the intervention arm of the trial have been trained on the content of the pathway.

No interventions will be implemented in the control group.

### Objectives

The objective of this cluster randomised controlled trial is to evaluate the effectiveness of pre-hospital and emergency clinical pathway for patients with suspected stroke in: improving early identification of stroke, promoting appropriate triage coding, achieving coherence between diagnosis, interventions and referral.

### Outcomes

The outcomes relate to organizational aspects (transportation to the appropriate hospital, accurate diagnosis and interventions, timely treatments) and will be measured as:

#### Primary outcome

- proportion of patients with a positive CT for ischaemic stroke and symptoms onset <= 6 hour admitted to the stroke unit

#### Secondary outcomes

- proportion of patients with a positive CT for hemorrhagic stroke and symptoms onset <= 6 hour admitted to a neurosurgical ward

- proportion of patients with a positive CT for hemorrhagic or ischaemic stroke or symptoms onset >6 hour admitted to the nearest hospital

- proportion of ICD9CM code for stroke in emergency setting confirmed in the hospital discharge data

- proportion of patients with stroke confirmed by CT results

- proportion of patients with ischaemic stroke receiving treatment within 6 hour of symptoms onset

All the outcomes will be reported at cluster level and will be cross-checked by the integration of data from the available information systems.

### Sample size

We calculated the sample size, i.e. the duration of recruitment, keeping into account the number of emergency calls and the number of emergency room admissions for suspect stroke.

For the ES the mean number of patients per 19 clusters was about 50 (see figure 2). The estimate percentage of correctly transferred patients, based on the Information System of Emergency Rooms in 2003, is 47%. We assumed an intra cluster correlation of ICC = 0.05. Under this assumption we decided 6 months duration, i.e. 25 patients per cluster, to obtain a power of 95% to detect a difference of 50% in rates between the two groups, i.e. reaching about 70% of correctly transferred patients in the treated group, with a = 0.05.

For the emergency rooms the mean number of patients per 14 clusters was about 110. The estimate of correctly transferred patients, based on the Information System of Emergency Rooms in 2003, is 14%. We assumed an intra cluster correlation of ICC = 0.05. Under this assumption and with a duration of 6 months, i.e. 55 patients per cluster, to obtain a power of 95% to detect a difference of 150% in rates between the two groups, i.e. reaching about 35% of correctly transferred patients in the treated group, with a = 0.05.

### Sequence generation and allocation

To avoid contamination due to personnel turnover, the entities were grouped into 20 clusters according to geographical nearness and personnel sharing procedures. Clusters were stratified according to their characteristics:

• Couples of one emergency service and one emergency room (usually working together in the same geographical area) (n°10)

• Groups of emergency services (n°6)

• Only emergency rooms (n°4).

Clusters were attributed sequential numbers and sample function of STATA 7 (StataCorp LP 2005) was used to generate random numbers. We utilized the Italian lottery extracted number of 6th November 2004 in Rome as seeds number for generating the random sequences.

### Data collection

To assess the impact of the adoption of the clinical pathway over the current practice, we will analyse the data currently available from the actual information systems and no additional information will be collected.

In this way, emergency health workers will not be charged with extra work deriving from registration of ad-hoc information, and they will be completely devoted to the implementation of the pathway itself.

The Agency of Public Health created and maintains the Information System of Emergency Rooms (ISER), the Stroke Surveillance System (SSS) and the Hospital Information System (HIS) sufficient to obtain data for measuring the outcomes. Moreover the Agency for Public Health has access to the Information System of ES 118 (IS118).

### Data analysis

• Baseline characteristics of the entities will be compared to ensure randomisation success;

• Analysis will be performed in an Intention to treat basis;

• Other analysis by protocol will be performed as a sensitivity analysis;

• Preliminary analysis will be performed after three month of the beginning of the observation.

All the analysis will be processed with SAS (version 8) and STATA 7 (StataCorp LP 2005).

### Ethics

The present protocol was designed following the indication of Helsinki adopted by the 18th World Medical Association General Assembly in June 1964 and following amended in 1975–2002 and clarified respectively for paragraph 29–30 in 2002 and 2004. The ethical committee of the Agency for Public Health approved the protocol with the document n.124 of 15 June 2004. The ethical committee was created according to Helsinki indications including, among others, a member of a patients' rights organization.

An informed consensus was signed by the responsible of each entity participating in the study who received a document containing all the relevant information and copies of the clinical pathway and the study protocol. No informed consensus will be requested to patients [7].

### Stopping rules

The nature of the intervention to be experimented prevented us from identifying specific stopping rules and we could only commit ourselves to submitting any possible problems to the ethical committee. The core of the pathway consists of transferring patients likely to benefit from complex interventions to specialized structures, rather than providing them with minimal assistance in the nearest hospital. This means that health workers belonging to experimental clusters may need to transfer patients for longer distance than they used to do. The risks we can foresee are unexpected events during transportation and troubles deriving from the unavailability of ambulances. Health workers have been warned to promptly report any kind of problems to the study coordinator.

## Discussion

The emergency and pre-hospital field has not been studied sufficiently in randomised controlled trials, likely reasons are organizational difficulties, critical conditions of patients and the related ethical problems [8].

We are therefore pioneering this kind of study in a difficult environment and we are prepared to face many obstacles. First, there are the changes in the network of the emergency services. During the last twelve months many new entities were created and others were suppressed generating problems in the identification of clusters. Lack of personnel and high turnover increase risk of contamination due to personnel sharing during the summer and the holydays.

Other problems have to do with the local law imposing that patients taken from the scene should be brought to the nearest hospital where the emergency physicians will determine the need (opportunity) to transfer elsewhere for appropriate cure. This procedure delay interventions and determines a very low percentage of cases directly admitted to the stroke unit: 14% of the ambulances transport (calls), and 47% of the emergency room admissions. However health workers participating in the experimentation expressed their worry about possible legal consequences of their decisions caused by the implementation of the protocol study, and this may represent an important obstacle to adherence.

Accuracy of data registration is crucial for process evaluation but our quality control system revealed that, even though a priori criteria for data collection are homogeneous, results are sometimes heterogeneous. Nevertheless, as misreporting is non differential the deriving underestimation may not severely affect results.

## Abbreviations

CME = continuous medical education

CT = computer tomography

ES = Emergency Service

ER = Emergency Room

ALS = Advanced Life Support

BLS = Basic Life Support

CHSS = Cincinnati pre-Hospital Stroke Scale

ICD9CM = International Classification Diseases 9^th ^revision Clinical Modification

ICC = intra cluster correlation

ISER = Information System of Emergency Rooms

SSS = Stroke Surveillance System

HIS = Hospital Information System

IS118 = Information System of ES 118

## Competing interests

The author(s) declare they have no competing interests.

## Authors' contributions

MF wrote the manuscript and assisted in the design of the study;

ADL contributed to the manuscript, ideated and coordinated the study;

PGR assisted in the cluster creation, sequence generation and allocation and analysis planning and contributed to the manuscript;

GL provided data analysis from the information systems and planning of information retrieval for the assessment of outcomes;

GG gave the input of the project, provided overview of all the steps of the study, and contributed to the manuscript final review.

All authors read and approved the final manuscript.

## Pre-publication history

The pre-publication history for this paper can be accessed here:


